# Neuroprotective, Neurogenic, and Amyloid Beta Reducing Effect of a Novel Alpha 2-Adrenoblocker, Mesedin, on Astroglia and Neuronal Progenitors upon Hypoxia and Glutamate Exposure

**DOI:** 10.3390/ijms19010009

**Published:** 2017-12-21

**Authors:** Magda M. Melkonyan, Lilit Hunanyan, Ali Lourhmati, Nikolas Layer, Sandra Beer-Hammer, Konstantin Yenkoyan, Matthias Schwab, Lusine Danielyan

**Affiliations:** 1 Department of Medical Chemistry, Yerevan state Medical University after M. Heratsi, 2 Koryun St., Yerevan 0025, Armenia; magda.melkonyan@meduni.am (M.M.M.); h-lilit@live.com (L.H.); 2 Department of Clinical Pharmacology, Institute of Clinical and Experimental Pharmacology and Toxicology, University Hospital of Tübingen, Auf der Morgenstelle 8, D-72076 Tübingen, Germany; ali.lourhmati@uni-tuebingen.de (A.L.); nikolas.layer@student.uni-tuebingen.de (N.L.); matthias.schwab@ikp-stuttgart.de (M.S.); 3 Department of Pharmacology and Experimental Therapy, Institute of Experimental and Clinical Pharmacology and Toxicology and ICePhA, University of Tuebingen, Wilhelmstr. 56, D-72076 Tübingen, Germany; sandra.beer-hammer@uni-tuebingen.de; 4 Biochemistry Department, Yerevan state Medical University after M. Heratsi, 2 Koryun St., Yerevan 0025, Armenia; konstantin.yenkoyan@meduni.am; 5 Dr. Margarete Fischer-Bosch Institute of Clinical Pharmacology, University of Tübingen, Stuttgart, Auerbachstr. 112, D-70376 Stuttgart, Germany; 6 Department of Pharmacy and Biochemistry, University of Tübingen, Auf der Morgenstelle 8, D-72076 Tübingen, Germany

**Keywords:** alpha adrenoblocker, mesedin, neurogenesis, astroglia, neurons, hypoxia, amyloid beta, glutamate

## Abstract

Locus coeruleus-noradrenergic system dysfunction is known to contribute to the progression of Alzheimer’s disease (AD). Besides a variety of reports showing the involvement of norepinephrine and its receptor systems in cognition, amyloid β (Aβ) metabolism, neuroinflammation, and neurogenesis, little is known about the contribution of the specific receptors to these actions. Here, we investigated the neurogenic and neuroprotective properties of a new α2 adrenoblocker, mesedin, in astroglial primary cultures (APC) from C57BL/6 and 3×Tg-AD mice. Our results demonstrate that mesedin rescues neuronal precursors and young neurons, and reduces the lactate dehydrogenase (LDH) release from astroglia under hypoxic and normoxic conditions. Mesedin also increased choline acetyltransferase, postsynaptic density marker 95 (PSD95), and Aβ-degrading enzyme neprilysin in the wild type APC, while in the 3×Tg-AD APC exposed to glutamate, it decreased the intracellular content of Aβ and enhanced the survival of synaptophysin-positive astroglia and neurons. These effects in APC can at least partially be attributed to the mesedin’s ability of increasing the expression of Interleukine(IL)-10, which is a potent anti-inflammatory, neuroprotective neurogenic, and Aβ metabolism enhancing factor. In summary, our data identify the neurogenic, neuroprotective, and anti-amyloidogenic action of mesedin in APC. Further in vivo studies are needed to estimate the therapeutic value of mesedin for AD.

## 1. Introduction

Targeting adult neurogenesis hampered by acute vs. chronic brain injuries and neurodegeneration has been considered a valuable therapeutic approach in a variety of brain disorders [1,2,3], including Alzheimer’s disease (AD) [4,5]. Among the strategies for increasing neurogenesis, cell-based therapy (CBT) is proposed to provide multifactorial neuroprotective and neurogenic mechanisms in neurodegenerative brain disorders. However, the survival and maintenance of the desired therapeutic phenotype of transplanted cells remain to create the major concern on the efficacy and sustainability of CBT [6,7,8]. 

Hypoxia and glutamate (Glu) excitotoxicity occur across various neurodegenerative diseases, including AD. Hypoxia is capable of upregulating cleavage of amyloid precursor protein (APP), and enhances amyloid beta (Aβ) production, both in vitro and in vivo, which in turn can reinforce glutamate neurotoxicity and facilitate AD pathogenesis [9,10,11]. Furthermore, oxidative stress, hypoxia, and Glu excitotoxicity were identified as the factors hampering the survival of cell therapeutics in the first days after transplantation [6,7,8]. Consequently, drug candidates that may affect not only endogenous neurogenesis, but also potentially influence the survival and differentiation of exogenously applied stem cells, may arguably address the unmet needs in drug and cell-based therapies of neurodegenerative disorders. Hereby, a search for the agents targeting receptor systems that are critically involved in the cellular defense against various pathological hallmarks of neurodegeneration may help to identify the powerful therapeutic candidates.

Among the receptor systems affected by AD pathology, adrenergic system is the one that increasingly gains attention, in the context of its involvement in memory formation, neurogenesis, and contribution to the progression of neurodegenerative changes upon AD. The neurotransmission of norepinephrine, released by the locus coeruleus neurons, is fulfilled via the α1, α2, and β adrenergic receptors located at postsynaptic neurons. Only α2 receptors are located, also presynaptically as autoreceptors, negatively regulating the release of norepinephrine. A dramatic degeneration of locus coeruleus has been observed in both AD patients and transgenic models of AD [12,13]. Furthermore, the noradrenaline precursor l-threo-3,4-dihydroxyphenylserine (l-DOPS) was shown to improve memory and to reduce Aβ plaque pathology by enhancing the levels of neprilysin (NEP) and insulin degrading enzyme in 5×FAD transgenic mice [14].

Among 1,4-benzodioxane derivatives, idazoxan, containing in the second position a five-membered heterocycle with two nitrogen atoms, is one of the most effective and widely used α2 adrenoblockers. However, because of its high degree of toxicity, the use of idazoxan is limited to experimental studies. In a search for new compounds combining α2-adrenergic blocking and anti-hypoxic properties, a 1,4-benzodioxane derivative, which contains five-membered heterocycle with two heteroatoms (nitrogen and sulfur) in the second position, has been identified as a potent selective α2 adrenoblocker [15]. 

**Figure 1:**
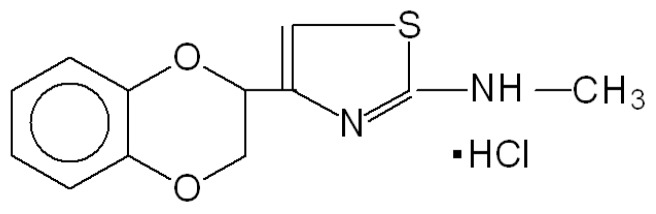
Chemical structure of 2-(2-methyl-amino-thiozolyl)-1,4-benzodioxane hydrochloride (mesedin) containing five-membered heterocycle with two heteroatoms (nitrogen and sulfur) in the second position.

This compound, 2-(2-methyl-amino-thiozolyl)-1,4-benzodioxane hydrochloride, named mesedin (Figure 1), was selected for further studies because of its pronounced properties for selectively and stably blocking the peripheral α2 adrenoreceptors, and its lower toxicity compared to the known structural analogs, beditin and idazoxan. Comparative acute toxicity study carried out on 106 mice revealed 50% and 14 times less toxicity for mesedin, than beditin and idazoxan, respectively [16].

The rationale of using mesedin as an α2 adrenoblocker, besides its less toxic properties in comparison with idazoxan, relies on the remarkable ability of mesedin to improve the survival of animals, memory decline, and anxiety symptoms, cerebral blood flow and tissue repair in a model of focal ischemia [17,18], as well as to protect against oxidative stress in a model of noise-induced stress [19]. These processes compromising the intactness of the brain tissue, including oxidative stress [20], impaired cerebral blood flow [21], memory deficits [22], and anxiety [23], are integral components of Alzheimer’s disease pathology. We hypothesized that the neurogenesis-inducing effect of mesedin may be at least the part of its regenerative effect, which was shown in a model of ischemia [24].

In view of the strong anti-hypoxic and antioxidative properties of mesedin [16], as well as previously demonstrated neurogenic and anti-amyloidogenic properties of the depletion [25], or the blockade of α2 adrenoreceptors, we sought to investigate the influence of mesedin on the survival and functional properties of neuronal precursors, astroglia, and newly generated neurons upon hypoxic and AD-like in vitro conditions.

## 2. Results

### 2.1. Mesedin Decreases the Lactate Dehydrogenase (LDH) Release from Astroglial Primary Culture

**Figure 2:**
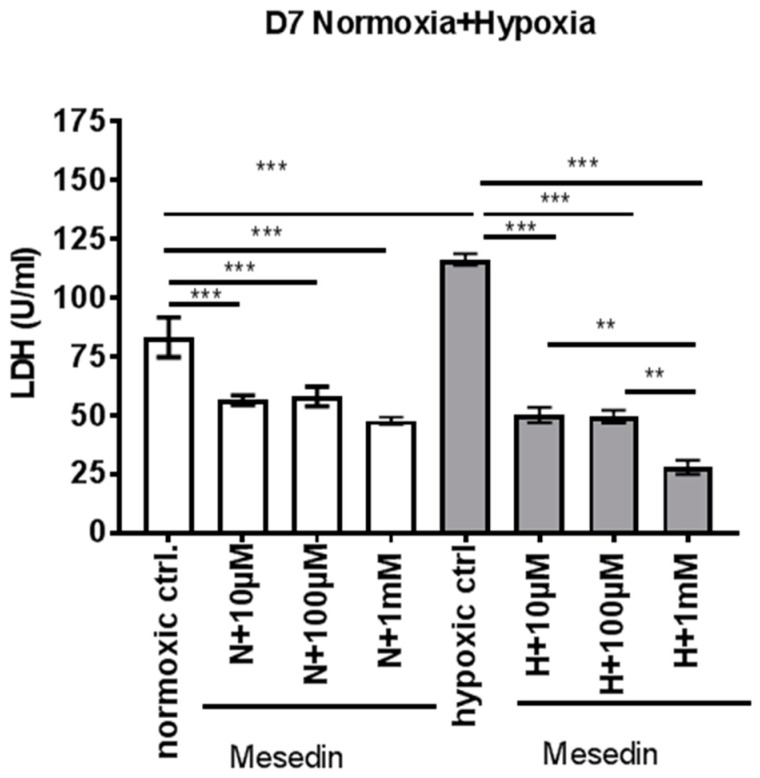
Lactate dehydrogenase (LDH) release from astroglial primary culture treated with mesedin upon normoxia and hypoxia. APC at day 7 in-vitro (DIV7) from newborn C57BL/6 mice (*n* = 6) were incubated for 48 h with ascending concentrations (10 µM, 100 µM, and 1 mM) of mesedin under normoxic (N, white bars) and hypoxic (H, grey bars) culture conditions and compared to respective controls without mesedin (normoxic and hypoxic ctrl.). The data presented as mean ± SEM were analyzed with one-way ANOVA and Bonferroni’s comparison test, ** *p* < 0.01, *** *p* < 0.001.

We assessed the lactate dehydrogenase (LDH)-release from astroglial primary culture (APC) at day 7 in vitro (DIV7) incubated with ascending concentrations of mesedin upon normoxic and hypoxic conditions. Hypoxia increased the LDH release by 33% (Figure 2, normoxic ctrl. compared to hypoxic ctrl).

Upon both normoxia and hypoxia, LDH decrease was achieved by all three concentrations used, where the highest concentration (1 mM) was the most effective in the hypoxic condition (Figure 2). In our further experiments, we used the lowest concentration of mesedin (10 µM), which turned to be efficacious in decreasing LDH release under both normoxic and hypoxic conditions in APC.

### 2.2. Mesedin Promotes Neuronal Commitment of Precursor Cells and Their Survival upon Hypoxia

**Figure 3:**
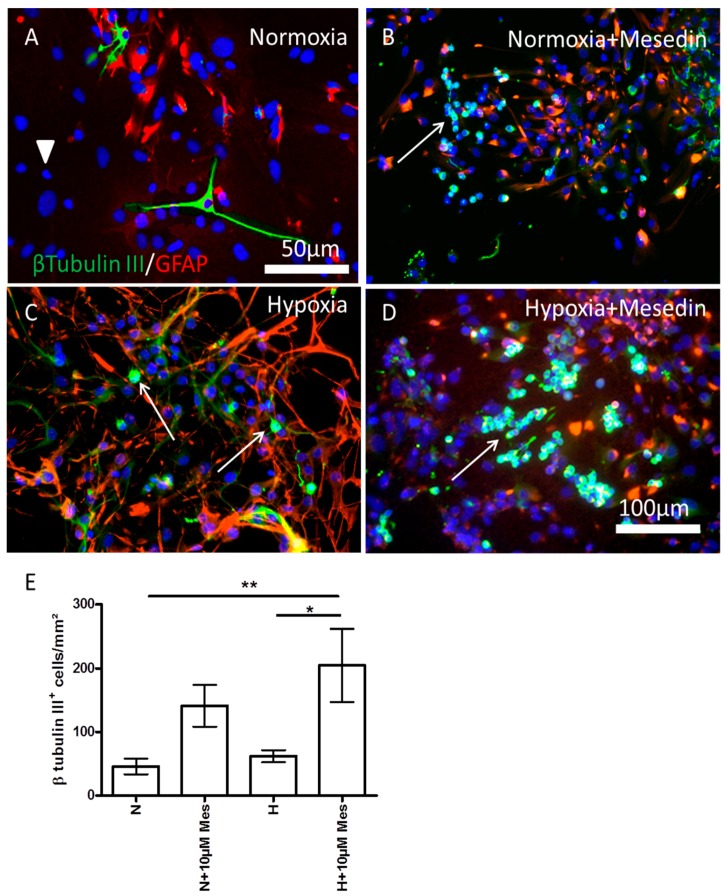
Neuroprotective effect of mesedin on DIV7 APC. The number of β-tubulin III-positive neurons (green) was assessed by double staining with glial fibrillary acidic protein (GFAP, red) in normoxic control (**A**), mesedin treated C57BL/6 APC upon normoxia (**B**), hypoxic control (**C**), and mesedin treated C57BL/6 APC upon hypoxia (**D**). Scale bar in (**A**): 50 µM, in (**B**–**D**): 100 µm. Arrowhead in A indicate the population of β-tubulin III and GFAP negative cells. Arrows in (**B**–**D**) indicate β-tubulin III-positive neurons/neuronal precursors. (**E**) Quantification of β-tubulin III-positive neurons/neuronal precursors upon normoxic (N) and hypoxic (H) culture conditions with 10 µM mesedin (N + Mes or H + Mes) in comparison to the respective controls (N vs. H). The cells were quantified from *n* = 5 coverslips, and normalized to mm^2^. The cell nuclei are counterstained with 4′,6-diamidine-2′-phenylindole dihydrochloride (DAPI, blue). Data are presented as mean ± SEM, and analyzed by two-way ANOVA with Bonferroni’s comparison test. * *p* < 0.05; ** *p* < 0.01.

**Figure 4:**
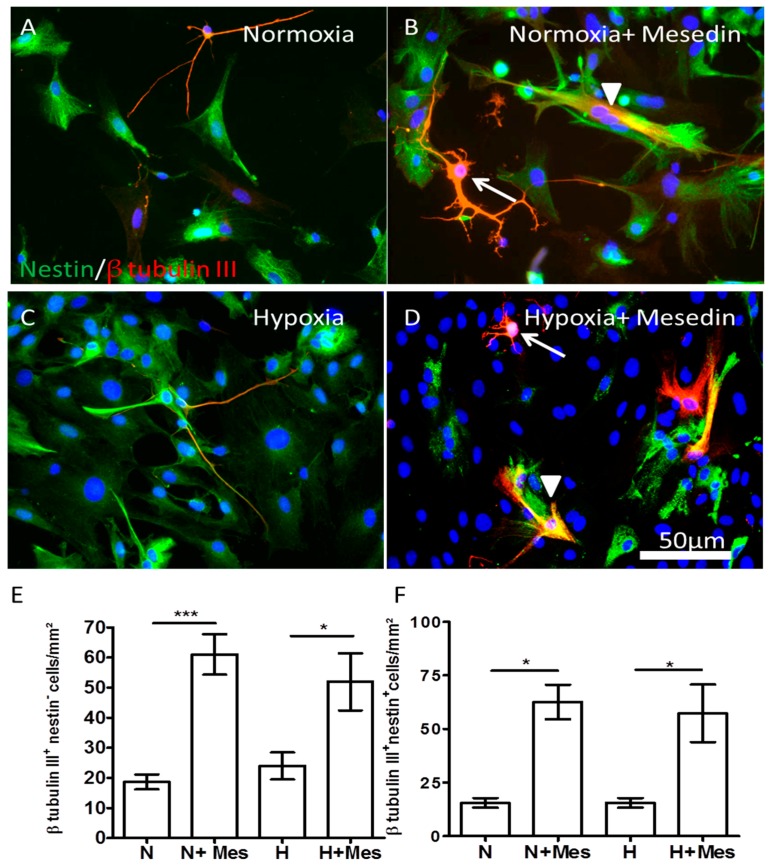
Mesedin enhances the survival of neurons and neuronal progenitors in APC. (**A**–**D**) Double staining of nestin (green) and of β-tubulin III-positive (red) neuronal progenitors, and neurons of 14 days old C57BL/6 APC under normoxic (**A**,**B**) and hypoxic (**C**,**D**) conditions. The cell nuclei are counterstained with DAPI (blue). Mesedin increases the survival and arborization of neurons (arrows in (**B**,**D**)), and the number of nestin^+^/β-tubulin III^+^ neuronal progenitors (arrowheads in (**B**,**D**)). Scale bar 50 µm. (**E**) Quantification of β-tubulin III-positive neurons upon normoxia (N) and hypoxia (H) in mesedin treated (N + Mes and H + Mes) and control (N and H) cultures. (**F**) Quantification of nestin^+^/β-tubulin III^+^ progenitors upon normoxia (N) vs. hypoxia (H) and mesedin treatment (N + Mes; H + Mes). The cells were quantified from *n* = 5 coverslips, and normalized to mm^2^. Data are presented as mean ± SEM, and analyzed by one-way ANOVA with Bonferroni’s comparison test. * *p* < 0.05; *** *p* < 0.001. Scale bar 50 µm.

Taking into consideration that mesedin was capable of decreasing the LDH-release also under normoxic culture conditions, hinting at a possible effect of mesedin on changes in different cell populations within the APC, we assessed the influence of mesedin on the population of β-tubulin III-positive neurons, and precursors expressing nestin and β-tubulin III (Figure 3 and Figure 4).

In the normoxic control, the APC contained mainly the population of GFAP^+^ astrocytes and neurons positive for β-tubulin III (Figure 3A). The population of GFAP and β-tubulin III-negative (presumably precursor) cells in normoxic control was detectable only with 4′,6-diamidine-2′-phenylindole dihydrochloride (DAPI) nuclear staining (Figure 3A arrowhead). This population nearly disappears in mesedin treated APC upon normoxia and hypoxia (Figure 3B,D). Since other cell types in APC (microglia, endothelial cells, and mature oligodendrocytes) cannot give rise to neurons, the population of cells negative for β-tubulin III and GFAP is most likely to be attributed to the population of precursor cells that switch to β-tubulin III (arrows in Figure 3B,D), and/or GFAP-positive phenotype upon mesedin treatment. Hypoxia increased the population of differentiated GFAP^+^ astrocytes, while the content of β-tubulin III-positive cells was restricted to a very few immature (judging from their shape) neuronal precursors (arrows in Figure 3C). Mesedin treatment of hypoxic cultures led to a remarkable increase of this β-tubulin III-positive population (arrow in Figure 3D). Quantification of β-tubulin III-positive cells displayed a significant increase in population of neuronal precursors/young neurons treated with mesedin upon both normoxic and hypoxic culture conditions (Figure 3E).

We further addressed the question whether mesedin can also enhance the population of neural precursor cells and influence their commitment to the neuronal lineage in advanced stages of astroglial differentiation. Therefore, we used APC at DIV14, and assessed the number of β-tubulin III/nestin-positive cells upon normoxic and hypoxic culture conditions. Indeed under both normoxia and hypoxia, in comparison with the respective control condition (Figure 4A,C), mesedin-treated culture contained a higher number of neuronal progenitors (arrowheads in Figure 4B,D) and neurons (arrows in Figure 4B,D), as shown by immunocytochemistry.

Quantification of β-tubulin III^+^/nestin^−^ neurons (Figure 4E) and β-tubulin III+/nestin+ progenitors in DIV14 APC (Figure 4F) revealed also a higher number of both cell populations upon mesedin treatment. In addition, a pronounced tendency for neuronal arborization was observed upon mesedin treatment (arrows in Figure 4B,D).

### 2.3. Mesedin Promotes the Expression of Interleukine-10, Postsynaptic Density Protein 95, and Choline Acetyltransferase in APC upon Hypoxia

**Figure 5:**
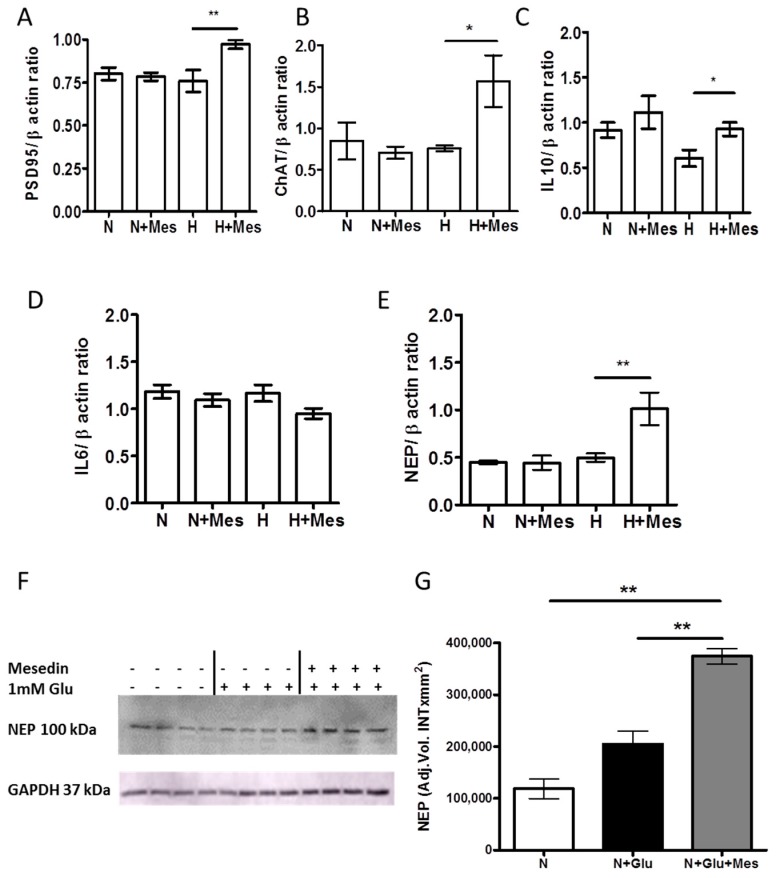
The impact of mesedin on the cholinergic, synaptic, and inflammatory markers in wild type APC and Aβ degrading function of 3×Tg-AD APC. (**A**–**E**) qPCR analyses of postsynaptic density protein 95, PSD95(**A**), choline acetyltransferase, ChAT (**B**), Interleukine-10 (**C**), Interleukine-6, IL-6 (**D**), and neprilysin, NEP (**E**) upon normoxia (N), hypoxia (H) with 10 µM mesedin (N + Mes; H + Mes) vs. respective controls (N; H). (**F**) Representative Western blot and (**G**) densitometric analysis of NEP in APC from two-month-old 3×Tg-AD mice upon normoxia (N) with and without glutamate (Glu), and 10 µM mesedin (*n* = 4). Glycerinaldehyd-3-phosphat-Dehydrogenase (GAPDH) served as loading control. Data are presented as mean ± SEM and analyzed by one-way ANOVA with Bonferroni’s comparison test. * *p* < 0.05; ** *p* < 0.01.

Acknowledging the ability of mesedin to expand the population of neuronal progenitors and neurons in vitro, and to protect them upon hypoxic conditions, we examined whether it can also enhance the synaptic activity and production of neurotransmitters in newly generated neurons and progenitors. Notably, only upon hypoxia, mesedin increased the expression of postsynaptic density ptrotein (PSD95) and choline acetyltransferase (ChAT) mRNA (Figure 5A,B). Extrapolating our in vitro results, showing neuroprotective and ChAT-/PSD95-increasing effects of mesedin on the brain primary culture to neurodegenerative conditions, it is tempting to speculate that mesedin may be a valuable candidate for slowing down neurodegenerative processes, leading to reduced neurogenesis and decreased cholinergic activity in Alzheimer’s pathology [4,26]. Thus, we addressed the question whether mesedin may exert its neuroprotective and neuroregenerative features by affecting neuroinflammatory processes. Among the determinants of the inflammatory response to the AD pathology, Interleukine-6 (IL-6) and IL-10 are within a range of those cytokines mostly reported to display the direct or inverse correlation with neurodegenerative changes in AD [27,28,29].

The qPCR analysis of IL-10 and IL-6 mRNA in cultures treated with mesedin upon normoxia and hypoxia revealed the increase in IL-10 mRNA upon hypoxia + mesedin (Figure 5C), while IL-6 mRNA remained unchanged in all tested conditions (Figure 5D).

Given that IL-10 promotes the degradation of Aβ in mononuclear phagocytes by influencing the expression of Aβ degrading enzymes [30], we tested the effect of mesedin on the expression of neprilysin (NEP), the Aβ-degrading enzyme that shows a reliable correlation with Aβ accumulation in the brain [31,32]. Neprilysin mRNA expression was significantly upregulated in mesedin-treated cultures, only upon hypoxia (Figure 5E).

### 2.4. Mesedin Enhances the Aβ Metabolism by Astroglia from 3×Tg-AD Mice

As a consequence of our findings on mesedin’s neuroprotective and neuroregenerative effects, as well as its capacity to influence the neuroinflammation and the Aβ-degrading enzyme in normal APC (upon hypoxic and normoxic culture conditions), we further tested whether these features are relevant to cultures exerting Alzheimer’s-like pathology. Therefore, APC from 3×Tg-AD mice were isolated and cultured in normoxic conditions. To generate the in vitro AD-like condition that comes closer to the in vivo AD-like pathology, the addition of glutamate (Glu) was considered to mimic the high extracellular content of Glu in the brain affected by AD [33]. As a first step, we assessed the expression of neprilysin upon exposure to Glu and mesedin by Western blot and densitometric analyses (Figure 5F,G). Exposure of Glu displayed a tendency to increase neprilysin in comparison with the control without Glu, while the administration of mesedin upon Glu exposure significantly increased neprilysin when compared to controls with and without Glu (Figure 5F,G).

**Figure 6:**
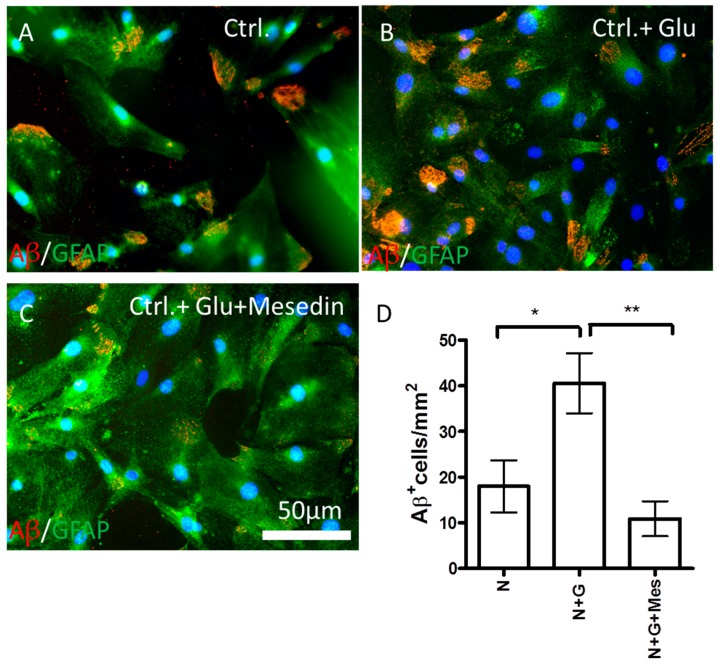
Mesedin reduces intracellular Aβ in 3×Tg-AD astroglia. Double immunostaining of Aβ by 6 E10 antibody (red) and GFAP (green) in APC from two-month-old 3×Tg-AD mice upon normoxic control (ctrl.) conditions without and with Glu ((**A**) vs. (**B**) respectively) and administration of 10 µM mesedin (**C**). (**D**) The Aβ^+^ cells were quantified from *n* = 5 coverslips and normalized to mm^2^. The cell nuclei are counterstained with DAPI (blue). Data are presented as mean ± SEM and analyzed by one-way ANOVA with Bonferroni’s comparison test. * *p* < 0.05; ** *p* < 0.01. Scale bar 50 µm.

Whether the mesedin-induced increase in neprilysin will lead to changes in intracellular Aβ accumulation was tested by immunocytochemical analyses of Aβ content in the 3×Tg-AD APC cultures with and without Glu exposure (Figure 6). Glu exposure to the 3×Tg-AD APC led to a higher positivity of Aβ in GFAP+ astroglia (cf. Figure 6A,B). A significant decrease in intracellular Aβ content was seen upon administration of mesedin in comparison with controls exposed to Glu, as it was shown by immunocytochemistry (cf. Figure 6B,C) and the quantification of Aβ-positive cells (Figure 6D).

### 2.5. Hypoxia and AD-Like Condition Increase the Expression of α2 Receptor

**Figure 7:**
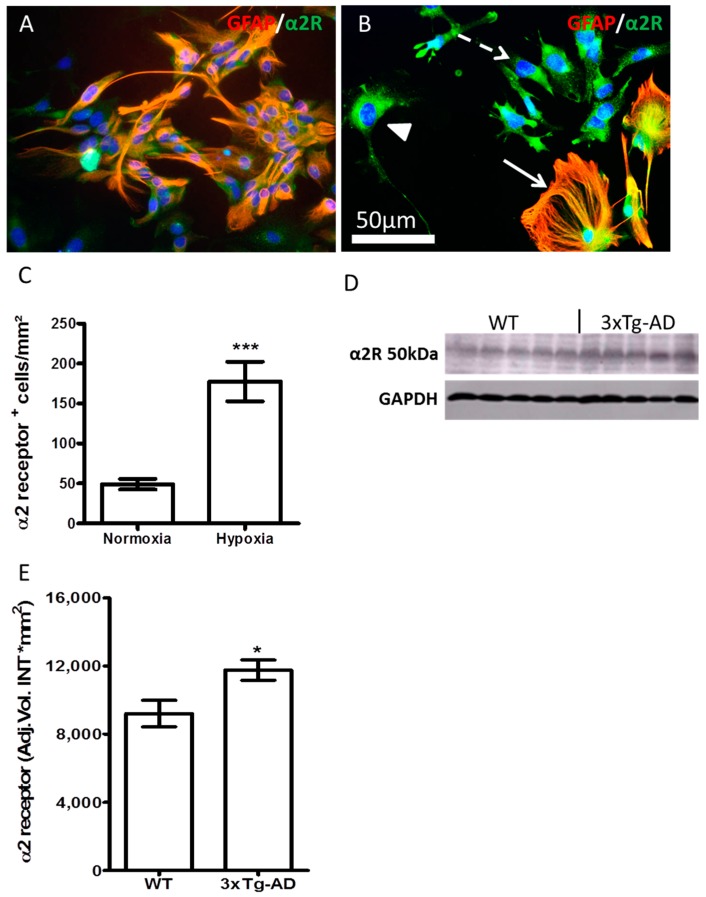
Increased expression of α2 receptor upon hypoxia and AD-like culture condition. (**A**) APC (DIV7) from the brains of new born C57BL/6 stained for GFAP (red) and α2 receptor (green); (**B**) Increased α2 receptor (green) in GFAP+ (red) astroglia (arrow), neurons (arrowhead), and fibroblast-like progenitors (dashed arrow) upon hypoxia; (**C**) Quantification of α2 receptor-positive cells in DIV7 APC from the brains of C57BL/6 newborn mice. (**D**) Western blot analysis of α2 receptor (α2R) from the brain homogenates of 14-month-old 3×Tg-AD and wild type (WT) C57/BL6 mice. (**E**) Densitometric analysis of (**D**). Data are presented as mean ± SEM (*n* = 5) and analyzed by Student’s *t*-test. * *p* < 0.05; *** *p* < 0.001. Scale bar in (**B**) is 50 µm.

Hypoxia enhanced the expression of α2 receptor in DIV 7 APC (cf. Figure 7A,B). A higher expression of α2 receptor was seen in astroglia (Figure 7B, arrow), and as judged from the morphology of GFAP-negative cells, in neuronal progenitors (Figure 7B dashed arrow) and neurons (Figure 7B arrowhead).

In aged (14-month-old) 3×Tg-AD mice, the expression of α2 receptor in the brain homogenates was upregulated in comparison to the age-matched WT controls, as shown by Western blot (Figure 7D) and densitometric analysis (Figure 7E).

### 2.6. Increased Expression of Synaptophysin upon Mesedin in Astroglia and Neurons from 3×Tg-AD APC

**Figure 8:**
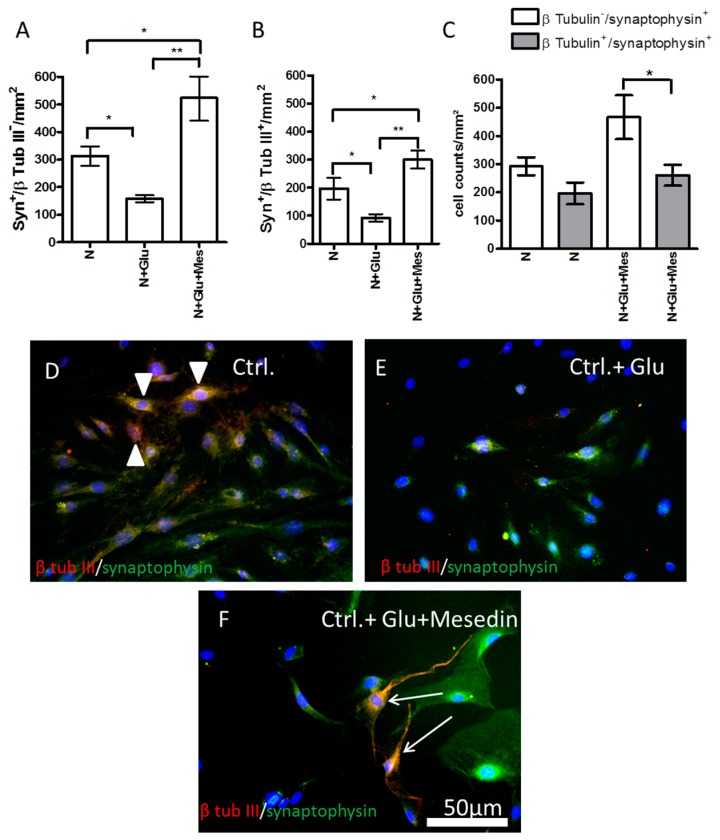
Mesedin enhances the survival of neurons and the expression of synaptophysin in 3×Tg-AD APC. (**A**) Quantification of synaptophysin^+^/β-tubulin III^−^ astroglia in APC from two-month-old 3×Tg-AD mice upon normoxic control (N) conditions, without and with Glu, and administration of 10 µM mesedin (Mes). (**B**) Quantification of synaptophysin^+^/β-tubulin III^+^ neurons in 3×Tg-AD APC. (**C**) Comparative analysis of synaptophysin^+^/β tubulin III^+^ neurons and synaptophysin^+^/β-tubulin III^−^ in 3×Tg-AD APC. The cells were quantified from *n* = 5 coverslips and normalized to mm^2^. Data are presented as mean ± SEM, and analyzed by one-way ANOVA with Bonferroni’s comparison test. * *p* < 0.05; ** *p* < 0.001. (**D**–**F**) Double immunostaining of synaptophysin (green) and β-tubulin III (red) in 3×Tg-AD APC. The cell nuclei are counterstained with DAPI (blue). Synaptophysin^+^/β-tubulin III^+^ neurons exert an immature round shape in normoxic control condition (arrowheads in (**D**)). Mesedin prevents the Glu-induced death of β-tubulin III^+^ neurons in 3×Tg-AD APC and enhances their maturation (arrows in (**F**)). Scale bar 50 µm.

Keeping in mind the effect of mesedin on the synaptic marker and the protection of neuronal population against hypoxia in the wild type APC (Figure 3 and Figure 4), we tested the influence of mesedin on the survival of β-tubulin III^+^/synaptophysin^+^ neurons and β-tubulin III−/synaptophysin+ astroglia in 3×Tg-AD APC upon exposure of Glu (Figure 8). Both astroglia and neurons responded to mesedin (upon Glu exposure) with increased synaptophysin reactivity in comparison to the controls with or without Glu (N and N + Glu vs. N + Glu + Mes in Figure 8A,B). Within both cell types (β-tubulin III-positive and -negative cells) the population of synaptophysin-positive cells under normoxic control conditions was nearly equally small (Figure 8C). In contrast, in mesedin-treated cultures, the population of synaptophysin-positive (presumably) astrocytes was larger than synaptophysin-positive neurons (Figure 8C). Interestingly, mesedin was capable of not only increasing the population synaptophysin-positive cells, but also protecting neurons (arrows in Figure 8F) against Aβ- and Glu toxicity in the 3×Tg-AD APC, when compared with the control exposed to Glu that nearly lacked neuronal population (Figure 8E,F). Mesedin appeared also to promote the differentiation of neurons reflected by morphological changes in β-tubulin III+ population between the control without Glu and mesedin + Glu treated APC. The majority of β-tubulin III^+^ cells in the control displayed a round or spindle-like shape (Figure 8D arrowheads), while in the mesedin treated group, the β-tubulin III^+^ population exerted a more mature neuronal morphology (Figure 8F, arrows).

## 3. Discussion

In view of controversial data on the protective vs. deleterious effects of the α2 blockade in the brain during hypoxia and Glu-excitotoxicity-associated neurodegeneration, such as AD [25,34], we sought to investigate the direct influence of the α2 antagonist mesedin on the survival, differentiation, and functional markers of neuronal precursors and astroglia upon hypoxia. Further, this study aimed also to investigate the protective effects of α2 antagonist mesedin on the neuronal precursors, neurons, and astroglia upon hypoxia and Glu toxicity in vitro. Our data show the capacity of the α2 antagonist mesedin to protect neurons against hypoxia and Glu-toxicity, to induce neuronal commitment of the precursor cells, to promote the cholinergic differentiation and the expression of synaptic markers in neuronal precursors, as well as to increase the Aβ-metabolizing function of astroglia in the hypoxia, and AD-like in vitro models.

A remarkable decrease in LDH-release in young (DIV7) APC upon normoxia/hypoxia and mesedin administration shown here reflects the protection of not only astrocytes, but also neural progenitors. DIV7 APC containing astroglia and nestin-positive precursor cells were proven to be less vulnerable to the hypoxic conditions, as assessed by LDH-release, than DIV21 culture containing mainly differentiated mature astroglia [35]. A lower content of LDH in the cell culture supernatant in DIV7 APC under non-toxic normoxic conditions and administration of mesedin may be explained by slowed differentiation of astroglia, and an increase in the number of nestin^+^/β-tubulin III^+^ precursor cells.

In the central nervous system (CNS), α2 receptors are widely expressed in astroglia and different types of neurons [36,37,38]. To prove the protective features of α2 blockade on the population of neurons and neuronal progenitors, we further assessed the survival of β-tubulin III-positive (nestin-positive and -negative) cells under hypoxic condition. Our data showing a mesedin-induced increase in the population of neuronal progenitors and young neurons, are in line with previous reports demonstrating an in vivo increase in survival of the newborn neurons in the dentate gyrus and olfactory bulb of adult rats upon α2 blockade [39,40]. Along with the findings by Rizk et al. [40], our results suggest that the increase in the number of neuronal progenitors and neurons is the result of their better survival upon α2 blockade rather than their proliferation, since even under physiologic conditions, α2 blockers can improve the survival of neural progenitors in vivo [40] and in vitro, as shown here by assessment of the LDH-release and quantification of β-tubulin III/nestin-positive cells upon normoxic conditions in APC. As a consequence of their neurogenesis-improving effect, α2 receptor antagonists have been proposed to be valuable drug candidates for neurodegenerative disorders, especially Alzheimer’s disease, where a decline in neurogenesis has been shown in a transgenic model of AD and in patients with AD [4,5]. Another hint to the advantages of the central α2 receptor blockade for the treatment of AD has been provided by a study showing a prominent memory loss after administration of α2 receptor agonist oxymetazoline [41]. In addition, a beneficial effect of α2 receptor blockade on the cognitive function has been proven in different studies. For instance, the treatment of α2 blocker yohimbine led to an increase in fear conditioning and spatial memory improvement in rats [42,43]. Some of the reports proposed the involvement of α1 and β-adrenergic receptors in the beneficial effects of α2 blockade [40], since the blockade of presynaptic α2 autoreceptors increases the availability of norepinephrine to the postsynaptic α1 and β-adrenergic receptors, the stimulation of which were shown to induce neurogenesis, memory consolidation, and neuroprotection [44,45]. Further studies are needed to clarify whether a receptor-independent antioxidant action of norepinephrine (enhanced by α2-receptor blockade) on neurogenesis and the survival of neurons [46,47] are involved in the mesedin effects described here.

The mechanisms by which α2 antagonists may regulate the neurogenesis are yet unclear. One of the key factors that negatively regulate neurogenesis and the cognitive function is inflammation [48]. Among pro- and anti-inflammatory cytokines, the production of which is affected by AD pathology, leading to the potentiation of neurodegenerative changes, IL-6 and IL-10 are frequently described as key factors (for review see [49]). Our data demonstrate that mesedin is capable of increasing IL-10 expression in the wild type astroglial primary culture upon hypoxia. Thus, it can be suggested that α2 blockade by mesedin can provide a neuroprotective effect via increasing IL-10 that was proven to ameliorate the cognitive deficits and neuroinflammation, as well as to increase hippocampal neurogenesis in a transgenic AD mouse model [50]. Our data demonstrating the upregulation of IL-10 by mesedin only upon hypoxic condition may hint to the importance of the expression level of α2 receptors on astrocytes and neurons, that in turn can be regulated by the low oxygen content of the microenvironment [51], which occurs upon AD due to the vascular dysfunction and hypoperfusion [52,53]. 

The effect of mesedin on the cholinergic activity and synaptic formation and maturation via an increase in ChAT and PSD95 expression may be at least partially explained by the α2 blockade induced increase of neural growth factor, that in turn is capable of enhancing ChAT and PSD95 production [54,55]. The α2 blockade by mesedin appears also to increase the expression of the pre-synaptic marker of exocytosis, synaptophysin, in neurons, and astroglia from 3×Tg-AD APC, which is logically concordant with the notion that α2 agonists are involved in inhibition of synaptic vesicle exocytosis in hippocampal neurons [56]. Taking into account the synaptogenic function of astrocytes [57], it can be suggested that α2 blockade by mesedin provides the improvement of this function, acting (i) directly via its synaptophysin/PSD95-increasing effect on the neurons, and (ii) indirectly through the protection of astrocytes against hypoxia and Aβ cytotoxicity.

In view of the neuroprotective and neurogenesis inducing effects of α2 blockade underscoring its potential therapeutic features upon AD pathology, we further evaluated the influence of mesedin on Aβ metabolizing function of neural cells. Using the APC from the triple transgenic mouse model of AD exerting plaque and neurofibrillary tangles pathology [58], we demonstrate (1) upregulation of neprilysin, a key degrading enzyme of Aβ, in comparison to the controls with and without Glu; and (2) remarkable inhibition of glutamate-induced intracellular Aβ accumulation in astroglia. These findings are in line with the previous report by Chen et al. [25], demonstrating a decrease in amyloid plaque pathology in α2 adrenoreceptor deficient APP/PS1 transgenic mice. Given that presynaptic blockade of α2 adrenoreceptor increases the release of norepinephrine in the CNS, it can potentially increase the expression of neprilysin, not only in vitro, but also in vivo. This notion is supported by the study showing the increase in neprilysin in response to enhanced noradrenalin production by the norepinephrine precursor l-threo-3,4-dihydroxyphenylserine (l-DOPS) [14]. The in vivo effect of mesedin on Aβ degradation (and possibly its generation) remains to be elucidated in an in vivo transgenic mouse model of AD. Of note, is that 3×Tg-AD brain tissue exerts a pronounced expression of α2 adrenoreceptor in comparison to wild type controls. Whether mesedin is capable of slowing down the progression of AD-like neurodegeneration in transgenic mice will be elucidated in a follow-up study.

Summarizing our data, it can be suggested that the selective α2-adrenoreceptor blocker mesedin can be considered as a valuable drug candidate for neurodegenerative disorders, especially AD, which are associated with hypoxia, Aβ, and glutamate toxicity. Further in vitro and in vivo investigations should be undertaken to explore the exact signaling mechanisms behind the α2-adrenoreceptor blockade mediated effects on neurogenesis, neuronal maturation, neuronal and astroglial protection, and Aβ degrading function.

## 4. Materials and Methods

All animal experiments were approved by the local institutional committee of Animal Welfare (approval date 05/06/13) in Tübingen (Regierungspräsidium Tübingen, Tübingen, Germany) and performed in accordance with the German federal law regarding the protection of animals and “Guide for the Care and Use of Laboratory Animals” (National Institutes of Health publication 8th Edition, 2011).

### 4.1. Cell Culture

Primary astrocytes were isolated from the brains of newborn C57BL/6 N mice (Charles River Laboratories, Sulzfeld, Germany) or two-month-old 3×Tg AD mice (strain B6;129-Psen1tm1MpmTg(APPswe, tauP301L)1Lfa/Mmjax generated by Frank LaFerla (University of California—Irvine, Irvine, CA, USA) as described elsewhere [58]. The APC from newborn rodents contain approximately 70–90% astrocytes [59,60,61]. According to our previous data, this culture includes a small population (2%) of neurons [62], while approximately 50% of astrocytes were identified as nestin-positive [63].

Briefly, the animals were decapitated upon CO_2_ euthanasia. The brains were isolated and processed further on ice for cell culture preparation. The cells were mechanically dissociated, centrifuged, and cultured in DMEM (Dulbeccos modified Eagle’s medium) with 4.5 g/L glucose supplemented with 10% fetal bovine serum, 1% penicillin/streptomycin, and 1% pyruvate in a humidified atmosphere of 5% CO_2_ at 37 °C.

### 4.2. Immunocytochemistry

The APC from the brains of newborn C57BL/6N mice were plated on the coverslips in 6 cm dishes (500,000 cells per dish), and incubated until day 5 or 12. Thereafter, the medium was removed, and fresh medium without or with 10 µM mesedin was added and incubated 48 h under normoxia or hypoxia.

The APC from two-month-old 3×Tg AD mice (passage 20) were plated on the coverslips in 6 cm dishes at concentration of 500,000 cells per flask, and incubated for 48 h under normoxia. For the experiments with Glu exposure, the medium was then removed, and the cells were treated with or without 1 mM glutamate alone or with 10 µM mesedin (*n* = 4), and incubated for 24 h under normoxia.

The cells were then washed with phosphate-buffered saline (PBS), and fixed with 4% formaldehyde for 10 min at room temperature (RT), and washed for 2 × 5 min with PBS. After fixation, the cells were incubated with primary antibodies, mouse monoclonal anti-β-III tubulin (1:200, abcam, Cambridge, UK), rabbit polyclonal anti-nestin (1:400, BD Biosciences, Heidelberg, Germany), rabbit polyclonal anti-GFAP (1:500, DacoCytomation, Glostrup, Denmark), rabbit polyclonal α2 receptor antibody (1:100; Sigma, Steinheim, Germany), rabbit anti-synaptophysin (1:400, Cell signaling, Cambridge, UK) and mouse monoclonal anti-amyloid β, 1-16 (1:500, 6E10, Covance, Princeton, NJ, USA). After washing twice with PBS, the cells were incubated for 1 h at room temperature (RT) in the dark with fluorescein isothiocyanate (FITC)-conjugated goat anti-rabbit IgG (1:100) or anti-mouse IgG (1:100), and cyanine (Cy)3-conjugated anti-mouse (1:800) or anti-rabbit IgG (1:800); all from Dianova (Jackson Immunoresearch, West Grove, PA, USA).

Thereafter, the cells were washed with PBS containing 0.05% Triton X-100 (Sigma), mounted using Vectashield Mounting Media containing 4′,6-diamidine-2′-phenylindole dihydrochloride (DAPI, Vector Laboratories, Burlingame, CA, USA), and evaluated by fluorescence microscopy with Olympus BX51 and analysis software (Olympus, Hamburg, Germany).

### 4.3. Western Blot Analysis

The APC from two-month-old 3×Tg AD mice (passage 2) were plated in 150 cm^2^ tissue culture flasks at a concentration of 600,000 cells per flask, and incubated for 3 weeks under normoxia. The medium was then removed, and the cells were treated with or without 1 mM glutamate alone, or with 10 µM mesedin (*n* = 4), and incubated for 24 h under normoxia.

After 24 h incubation, the medium was removed, and the cells were washed with sterile PBS before 2 mL of 0.05%trypsin- ethylenediaminetetraacetic acid (EDTA) was added for 5 min. at 37 °C. To stop trypsinization, 5 mL of DMEM containing FCS was added to the flask. Cells were collected and centrifuged by 4000× *g* at 4 °C for 8 min. The supernatant was aspirated, and the pellet was resuspended in lysis buffer (300 mM NaCl, 50 mM Tris, 2 mM MgCl_2_, 0.05% NP40, containing a “Complete Protease Inhibitor Tablet” from Roche Diagnostics), with 20 µL of lysis puffer per million cells, and freezing at −80 °C. Thereafter, the cells were centrifuged by 16,000× *g* at 4 °C for 20 min. The supernatant was removed for Western blot. 

Brain hemispheres from 14-month-old 3×Tg-AD and wild type C57BL/6 mice (*n* = 5) were homogenized in lysis buffer as described above.

Protein concentrations were estimated using the Lowry assay. For each lane, 50 μg of proteins were loaded and separated using 12.5% sodium dodecylsulphate (SDS) gel, and transferred to PVDF membranes by tank blotting. Membranes were blocked for 1 h at RT, and were then incubated at 4 °C overnight with antibodies against rabbit monoclonal neprilysin (1:1000, abcam) or rabbit polyclonal α2 receptor (1:750, abcam), and mouse monoclonal GAPDH (1:1000, Millipore, Eschborn, Germany; loading control). For visualization of antibody binding, membranes were incubated for 3 h at RT with Cy5 anti-rabbit (1:2000, GE Healthcare, Freiburg, Germany), and Qdot525 anti-mouse (1:1000, Life Technologies, Waltham, MA USA). Signal intensities were recorded using VersaDoc TM imaging system 4000 MP (Bio-Rad Laboratories GmbH, München, Germany). The densitometry for protein specific bands was done using Quantity One^®^ 1D analysis software (Bio-Rad, Hercules, CA, USA).

### 4.4. Cytotoxicity Assay

The measurement of lactate dehydrogenase (LDH) activity in the cell culture supernatants (*n* = 6) of 96-well microtiter plates was performed following the protocol indicated in cytotox 96^®^ Non-Radioactive Cytotoxicity Assay (Promega Mannheim, Mannheim, Germany). The medium was removed at day 5, and fresh medium without or with 10 µM, 100 µM, and 1 mM mesedin was added, and the cells were incubated for 48 h under normoxia or hypoxia conditions.

For determination of LDH, 50 µL of the supernatant of each well was transferred to another 96-well microtiter plate, mixed with 50 µL substrate mix and incubated in the dark for 30 min at RT. To stop the enzymatic reaction, 50 µL stop solution was added to each well of the 96-well plate, and the absorbance was measured at 490 nm in the Tecan Sunrise plate reader (Tecan, Crailsheim, Germany). Quantification was done by external standardization with LDH concentrations in the range from 0–800 U/mL in DMEM (standard supplied in the test kit). The absorbance value of a culture medium control was used to normalize the values obtained from the samples.

### 4.5. Quantitative PCR

RNA isolation and qPCR: cells were lysed with RLT buffer, and for isolation, the RNA Mini Kit (Qiagen, Hilden, Germany) was applied according to the manual. Reverse transcription of RNA was performed with the Transcriptor High Fidelity cDNA Synthesis Kit (Roche Diagnostics Deutschland GmbH, Mannheim, Germany) according to the protocol provided. For quantitative PCR, 50 ng cDNA was used.

Quantitative PCRs were performed with a LightCycler 480 (Roche, Basel, Switzerland) with a primary 5 min denaturation step at 95 °C; 45 cycles with 10 s denaturation at 95 °C, 10 s annealing at 60 °C, and 10 s elongation at 72 °C; and a melting curve as final step with 5 s at 95 °C and 1 min at 65 °C. The PCR products were stored at 4 °C until analysis on a Qiaxcel (Qiagen). 

Primers for IL-6 were sense (5′-TGATGGATGCTACCAAACTGG-3′) and antisense (5′-TTCATGTACTCCAGGTAGCTATGG-3′), and generated a product of 96 bp. Primers for IL-10 were sense (5′-CAGAGCCACATGCTCCTAGA-3′) and antisense (5′-TGTCCAGCTGGTCCTTTGTT-3′), and generated a product of 79 bp. Primers for Nephrilysin were sense (5′-GGGAGGCTTTATGGAAGC-3′) and antisense (5′-CCGGATTTGTGCAATCAAGT-3′), and generated a product of 75 bp. Primers for PSD95 were sense (5′-GACGCCAGCGACGAAGAG-3′) and antisense (5′-CTCGACCCGCCGTTTG-3′), and generated a product of 96 bp. Primers for cholinacetyltransferase were sense (5′-GGTTCGGTGCGTAACAGC-3′) and antisense (5′-GCGATTCTTAATCCAGAGTAGCA-3′), and generated a product of 64 bp. Primers for β-actin were sense (5′-AAGGCCAACCGTGAAAAGAT-3′) and antisense (5′-GTGGTACGACCAGAGGCATAC-3′), and generated a product of 110 bp.

### 4.6. Statistical Analyses

All data were analyzed with GraphPad Prism, (GraphPad Software, San Diego, CA, USA). For multiple comparisons one- or two-way ANOVA, followed by Bonferroni post hoc analysis, were used. Wherever applicable, Student’s *t*-test was applied. The data are presented as mean ± SEM.
